# Perception and engagement in unprofessional behaviors of medical students and residents: a mixed-method study

**DOI:** 10.1186/s12875-023-02153-y

**Published:** 2023-09-18

**Authors:** Fatemeh Keshmiri, Mehdi Raadabadi

**Affiliations:** 1 Medical Education Department, Educational Developmental Center, Shahid Sadoughi University of Medical Sciences, Yazd, Iran; 2 National Agency for Strategic Research in Medical Education, Tehran, Iran; 3 Health Policy and Management Research Center, School of Public Health, Shahid Sadoughi University of Medical Sciences, Yazd, Iran

**Keywords:** Professionalism, Unprofessional, GP resident, Medical intern, Resident, Professional

## Abstract

**Background:**

The present study aimed to investigate perception and engagement in unprofessional behavior of residents and medical interns and explore the factors affecting their engagement in unprofessional behavior.

**Method:**

This study has an explanatory (quantitative-qualitative) mixed-method design. This study was conducted at Shahid Sadoughi University of Medical Sciences in 2022–2023. Participants, including residents and medical interns (n = 169), were entered by stratified random sampling. A survey was conducted in the quantitative step. A by an unprofessional behavior in clinical practice questionnaire (29 items) was used. For each behavior, the participants were asked to report whether they (a) participated in the behavior and (b) stated that the behavior Is unprofessional. In the qualitative step, 17 participants contributed. The qualitative data were collected by semi-structured interviews and analyzed according to the conventional content analysis approach Graneheim and Lundman introduced.

**Results:**

The highest ratio of participants’ engagement in unprofessional behavior was reported in ‘failure to introduce yourself and nurses and physician assistants to the patient and his family’ (n = 145 (85.8%)). The results showed the proportion of participants who engaged in unprofessional behavior more than those who did not participate. There were associations between participants’ engagement in each behavior and their perception of that particular behavior as unprofessional. (p = 0.0001). In the following behaviors, although the participants acknowledged that these behaviors were unprofessional, those who participated in the unprofessional behaviors were significantly more than those who did not participate: failure to comply with clinic regulations and policy (p = 0.01), eating or drinking in the hallway of the clinic (p = 0.01), medical negligence in duties in the clinic setting (p = 0.04) and failure to perform duties in teamwork (p = 0.04). The qualitative results were explored in a theme entitled “internalized unprofessional culture,” including three categories “encouraging contextual risk factors towards unprofessionalism,” “suppressing of unprofessionalism reporting,” and “disbelieving professionalism as a key responsibility.”

**Conclusion:**

The results indicated that most participants engaged in unprofessional behaviors. The findings resulted from the internalized unprofessional culture in the workplace. The findings showed that engagement in unprofessional behaviors resulted from personal and systemic factors. The weakness of responsibility recognition and identity formation as a professional facilitated the engagement in unprofessional behaviors at the personal level. Furthermore, systemic factors including the contextual risk factors (such as deficiency of explicit and hidden curriculum), and the suppression of unprofessionalism reporting mechanism as a hidden factor played an important role in normalizing unprofessional behavior and promoting engagement in unprofessional behaviors among the participants. Recognition of the nature and extent of students’ unprofessional behaviors facilitates educational discussion among teachers and students in this field. The results might assist to establish an assessment system and feedback mechanism to solve the problem of the “failure to fail” problem. In addition, these results provide medical educators insights into the development of professional courses that equip learners with adherence to professionalism and coping skills to deal with unprofessionalism in the healthcare system.

## Introduction

The development of professional behavior has been introduced as a requirement for improving patient safety and patient care outcomes [1]. Professionalism is defined as “an ideal-typical set of characteristics for a profession; specialized knowledge/skills, self-control/regulation, the division of labor, defined training pathway, monopoly, and a code of ethics” [2]. Professionalism comprises the domains of altruism, respect, error disclosure, responsibility, confidentiality, and integrity [3]. The main goal of professionalism is to establish trust between patients and healthcare providers [4]. Professionalism has been recognized as a key capability in medical curriculums [5, 6]. A growing body of literature has confirmed the negative impact of unprofessional behaviors of healthcare workers on patient safety and organizational outcomes. Most medical complaints were reported due to the unprofessional behavior of the healthcare workers compared to their deficiency of knowledge and skills [7, 8]. The results of Bahaziq’s study showed a positive relationship between unprofessional behaviors and their undesirable consequences, such as patients’ endangerment and dissatisfaction [7].

A growing body of research has shown that the prevalence of unprofessional behaviors is warranted to explore how the phenomenon of unprofessional behavior expands among all workers’ groups across different healthcare contexts [9–12]. Westbrook and colleagues showed that 39% of staff reported experiencing one or more unprofessional behaviors [13]. They indicated that the workers’ perceptions impacted by the organizational factors associated with reporting and reducing these behaviors [13]. The prevalence of unprofessional behaviors in the medical educational systems was recognized as an issue of the hidden curriculum that negatively affects learners’ professional identification [4]. Exploring professional dilemmas and unprofessional behaviors is an essential step in recognizing educational problems in this era.

Professional behavior as a values-based concept is influenced by institutional, local, and international codes of conduct [14]. As well, professionalism was influenced by the cultural components of the healthcare system [15]. Thus, assessment and monitoring of the influenced factors in different components of a system are required. The establishment of unprofessional culture as the main issue in education and healthcare systems requires consideration by managers and leaders. Yavari and colleagues stated despite all efforts to enhance professional behavior among medical trainees, unfortunately, information from medical schools around the world endorses the predominance of unprofessional behaviors of medical students. It is required to address reasons for failing to minimize unprofessional performance among medical students in different countries with different contexts and cultural components [16].

In our context, there was no educational program or planned mechanism that focused on professionalism in explicate and hidden curriculum. As well, there was no planned assessment system for the professional and unprofessional behavior of learners and workers. The results of different studies in this context confirmed the issues of professionalism among practitioners and learners in the healthcare system [16–19]. Parizad and colleagues explored the nurses’ experiences of professional communication between colleagues in the emergency department. Their results explored the individual and collective unprofessional behavior among professionals. They acknowledged staff attitudes and behaviors were inconsistent with expectations of professional behavior and practice [19]. Yavari and colleagues conducted a study aimed to explain the challenges of Iranian medical students in providing professional behavior. Their results classified the obstacles to professional behavior into three main categories: problems related to the educational system, problems related to society, and problems related to students themselves. Their results acknowledged various personal, social, and educational factors that created and expanded unprofessional behaviors among medical students. Thus, it is essential to further study the description of the phenomena of unprofessional behavior and reasons to develop a comprehensive approach to solving the problem [16]. Recent studies have acknowledged the need to explore and categorize unprofessional behavior in healthcare contexts [15, 20, 21]. The use of mixed method methodology was suggested to allow addressing more complex research questions using a combination of quantitative and qualitative data in studies of the professional field [22–24]. The quantitative findings explain the prevalence of unprofessional behaviors in different professions and explore the reason and risk factors of unprofessional behaviors in the qualitative results. The mixed-method studies integrated and triangulated the findings from both quantitative and qualitative phases, which enriches the study by better explaining the phenomena in the investigated context and confirming and refuting results on how the findings of the two phases shared similarities and differences [22].

The present study aimed to investigate perception and engagement in unprofessional behavior among medical interns and residents and explore the factors affecting their engagement in unprofessional behavior.

## Method

The current study has an explanatory mixed-method design. “An explanatory sequential mixed methods design consists of first collecting quantitative data and then collecting qualitative data to help explain or elaborate on the quantitative results. The rationale for this approach is that the quantitative data and results provide a general picture of the research problem; more analysis, specifically through qualitative data collection, is needed to refine, extend, or explain the general picture.” [25] In this study used the explanatory mixed-method design to investigate the participants’ unprofessional behavior as a general picture of the research problem in the quantitative step and explore the reasons for their behaviors in the qualitative step. The quantitative step aimed to investigate the participants’ perception and engage in unprofessional behavior through a survey, and the qualitative step was conducted to explore the participants’ experiences with the factors affecting their engagement in unprofessional behavior.

Setting: This study was conducted at Shahid Sadoughi University of Medical Sciences in 2022–2023.

### Participants

Quantitative step: The population included all residents and medical interns (n = 272) of Shahid Sadoughi University of Medical Sciences. The inclusion criterion was the contribution in at least a 6-month of work in clinical units. The sample size was calculated (p = 0.5, q = 0.5, z = 1.96, d2 = 0.05, N = 272) and 169 participants were entered into the study. The participants’ groups (residents and medical interns) were entered by stratified random sampling. Stratified random sampling was used when a population was divided into smaller subgroups based on members’ shared attributes or characteristics [26, 27]. In this study, the participants were divided into subgroups of residents and medical interns.

Qualitative step: The participants who achieved the lowest and highest score in the quantitative phase were contributed by maximum variation sampling. The participants (n = 17) include nine medical interns (53%) and eight residents (47%) who participated in the study. The mean ± SD age of participants was 30 ± 3.3.

### Data collection tools and analysis method

The questionnaire was organized into three parts including (1) demographic characteristics (age, gender, academic course), (2) binary questions related to their previous information about professional ethics, and (3) a 29-item questionnaire of ‘unprofessional behaviors in clinical practice.’ The participants were asked to report whether they (a) participated in the behavior and (b) stated that the behavior Is unprofessional or not. The scoring of questionnaires was binary (yes = 1 and no = 0)). The range of scores was from 0 to 54. The validation of the questionnaire was confirmed in Jamalabadi and Ebrahimi’s study (Cronbach’s alpha = 0.79 and test-retest reliability, ICC = 0.80) [28]. In the present study, the internal consistency of the questionnaire was approved by Cronbach’s alpha = 0.82.

For data collection, the objective of the research was explained to the participants and written informed consent was obtained from them. The participants were asked to fill out the paper-and-pencil survey. The anonymous questionnaire was collected by the researcher (M.R.) in the teaching hospitals.

#### Quantitative data analysis

Data were summarized by descriptive statistics (mean, SD, frequency, and percentages). The association between participation in behavior and perception of that behavior as unprofessional was tested using the McNemar Test. In addition, we have compared the categorical variable such as participants’ gender and groups by chi-square test (χ2). Statistical significance is defined as p = 0.05.

### Qualitative phase

In the present study, the participants’ experiences related to the factors affecting their engagement in unprofessional behavior were explored through the conventional content analysis approach introduced by Graneheim and Lundman [29].

Data was collected through individual and semi-structured interviews. A trained interviewer (M.R., MSc in health professions education) conducted the interviews, and there was no relationship between the participants and the interviewer. The research’s purpose, the interview method, the individuals’ right to participate in the study, and the confidentiality of data were explained. Written informed consent was obtained from participants. The interviews were directed based on an interview guide to increase credibility. All interviews started with main questions such as “Could you explain your experiences regarding professionalism challenges that observed at the teaching clinic?”, “did you experience behavior that you thought neglected patients’ dignity or college? Moreover, “What are the factors that affect the occurrence of unprofessional behavior in your context?”. Some probing questions were asked to illuminate the participants’ responses. All interviews were recorded, and the interviewer made field notes during the interview. Each interview lasted between 35–45 minutes. The data collection process continued until a rich interpretation was obtained, and no new data emerged during the interviews (saturation of results). The data collection and analysis process was conducted in Persian and then translated into English for this paper.

Qualitative Data analysis: The data analysis was conducted according to the conventional content analysis approach, which Graneheim and Lundman introduced. The conventional content analysis approach includes exploring open coding, category, and theme [29].

In the data analysis step, the recorded interviews were transcribed. The interviews were read several times to attain a sense of the whole. The meaning units were extracted from the participants’ words and expressions, reflecting their experiences. “A meaning unit is words, sentences or paragraphs containing aspects related to each other through their content and context” [29]. After that, open codes emerged. The codes were noted in the text’s margins and then transferred to coding sheets. These codes were classified into a category based on their similarities and differences. The categories indicated a content group that shares a commonality [29]. After that, the theme was explored by linking the underlying meanings in categories, comparing, and contrasting the categories.

In this study, the data coding was conducted by two experienced persons in the qualitative content analysis research. An expert in qualitative research supervised the process. In cases of disagreement over the coding, discussions about the codes were continued until a consensus was achieved.

#### Trustworthiness

In this study, the criteria consisting of credibility, confirmability, transferability, and dependability described by Guba and Lincoln [30] were used to ensure trustworthiness. Semi-structured interviews, field notes, and lengthy engagement with the research topic were used to achieve the credibility of the data. Moreover, this study explored the research question from a variety of aspects, participants with various experiences contributed.

The extracted codes and categories were reviewed by the participants, the research team, and two experts. The texts and explored results were returned to the participants to ensure that extracted codes and categories were consistent with what they had experienced. (Member-check). The data analysis process was thoroughly examined by the research team (peer-check) and two experts in qualitative research. (Audit-review). This study’s steps of research, especially the data analysis, have been thoroughly documented. The transferability of the findings was achieved by the description of the context, participant characteristics, data collection, and data analysis process.

#### Ethical Considerations

The study was approved by the Ethics Committee at the National Agency for Strategic Research in Medical Education. Tehran. Iran. (ID: IR.NASRME.REC.1400.055). This study considered the principles of confidentiality of information, recording of interviews, and the right to withdraw from research.

## Results

In total, 169 participants contributed to the study, including 69 residents (40.82%) and 100 medical interns (59.17%). The mean (and ± standard deviation) age of participants was 28.42 ± 4.64, and 96 males (56.8%) and 73 females (43.2%) contributed to the study.

The results showed 84 participants (49.7%) were familiar with the meaning and application of professional ethics, 79 individuals (46.7%) passed a course in medical ethics before the study, and 62 contributors (36.7) reported having self-directed study about professionalism.

The highest ratio of participants’ engagement in unprofessional behavior was reported in ‘failure to introduce yourself and nurses and physician assistants to the patient and his family’ (n = 145 (85.8%)). (Table 1). Table 1 shows the perception and engagement in the unprofessional behavior of the participants.

**Table 1:** The perception and engagement in unprofessional behavior of the participants

Items	Number of Students participating in behavior				Students stating that behavior Is unprofessional				P-Value
	Non-Engagement		Engagement		Non-Engagement		Engagement		
	N	%	N	%	N	%	N	%	
1. Lack of maintaining medical dignity in their relationship, talking, dressing	69	40.8	100	59.2	67	41.4	95	58.6	0.4
2. Denial of any errors, mistakes and wrongdoing	73	43.2	96	56.8	70	44.3	88	55.7	0.21
3. Dishonest behavior in the workplace	63	37.3	106	62.7	60	37.7	99	62.3	0.45
4. Failure to comply with clinic regulations and policy	55	32.5	114	67.5	54	35.1	100	64.9	0.01*
5. Having personal conversations or making fun of students, other physicians, peers, or staffing the corridors of the clinic	52	30.8	117	69.2	45	29.8	106	70.2	0.29
6. Eating or drinking in the hallway of the clinic	56	33.1	113	66.9	55	35.7	99	64.3	0.01*
7. Medical negligence in duties in the clinic setting	56	33.1	113	66.9	54	35.3	99	64.7	0.04*
8. Lack of observance of discipline in medical work	55	32.5	114	67.5	47	33.6	93	66.4	0.37
9. Lack of commitment to be available and responsive when “on call”	58	34.3	111	65.7	50	33.6	99	66.4	0.36
10. Failure to perform duties in teamwork	44	26.0	125	74.0	41	28.7	102	71.3	0.04*
11. The use of alcohol or drugs in the workplace	118	69.8	51	30.2	47	32	10	68	0.14
12. Failure to report the risky and/or inappropriate behavior of a colleague (after approaching the individual)	52	30.8	117	69.2	46	32.6	995	67.4	0.17
13. Performing procedures without having sufficient skills (without supervision)	50	29.6	119	70.4	48	31.6	104	68.4	0.07
14. Lack of commitment to continuous learning	51	30.2	118	69.8	46	31.9	98	68.1	0.16
15. Disregard educational activities (e.g., arriving late to rounds for nonclinical reasons, skipping a lecture or seminars in which attendance is required)	52	30.8	117	69.2	47	31.5	102	68.5	0.37
16. Lack of self-assessment and refusal to accept and apply constructive critiques	43	25.4	126	74.6	38	26.2	110	73.8	0.38
17. Lack of equity and fairness in serving patients	47	27.8	122	72.2	43	28.1	110	71.9	0.52
18. Lack of acceptance of probable health risks him/herself in front of the patient’s	54	32.0	115	68.0	49	32.2	103	67.8	0.52
19. The lack of bearing difficulty and discomfort in responding to the medical needs of the patients	62	36.7	107	63.3	55	35	102	65	0.09
20. Play down feelings, needs and wishes of the patient	55	32.5	114	67.5	49	31.8	105	68.2	0.35
21. Lack of empathy and compassion with patients	60	35.50	109	64.5	56	36.4	98	63.6	0.32
22. Prefer their interests to the interests of the patient	62	36.7	107	63.3	48	36.1	85	63.9	0.45
23. Lack of commitment to patient privacy	73	43.2	96	56.8	67	44.4	84	55.6	0.26
24. Lack of respect for people’s religious and cultural differences	58	34.3	111	65.7	51	34.2	98	65.8	0.56
25. Addressing patient inappropriately	40	23.7	129	76.3	31	22.6	106	77.4	0.32
26. Lack of commitment to privacy of the patient-physician relationship	50	29.6	119	70.4	44	29.7	104	70.3	0.56
27. Not suggesting treatment options to patients who cannot afford them	53	31.4	116	68.6	44	31.9	94	68.1	0.46
28. Failure to maintain a professional boundary in relation to patients or colleagues	58	34.3	111	65.7	47	37.6	78	62.4	0.09
29. Failure to introduce yourself and nurses and physician assistants to the patient and his family	24	14.2	145	85.8	17	15.5	93	84.5	0.34

*p-value is significant

The results indicated there were associations between participants’ engagement in each behavior and their perception of that particular behavior as unprofessional. (p = 0.0001). In the following behaviors, although the participants acknowledged that these behaviors were unprofessional, those who participated in the unprofessional behaviors were significantly more than those who did not participate: (1) Failure to comply with clinic regulations and policy, (2) Eating or drinking in the hallway of the clinic, (3) Medical negligence in duties in the clinic setting and (4) Failure to perform duties in teamwork. There was a statistically significant difference in the ratio of perceptions about unprofessional behaviors in participants’ genders. (p = 0.006). 93.2% of female participants (n = 68) and 78.1% of males (n = 75) recognized the behaviors as unprofessional.

There was no statistically significant difference in the ratio of perceptions about unprofessional behaviors in participants’ groups (medical interns and residents). (p = 0.79). There was no statistically significant difference in the ratio of engagement in unprofessional behaviors in participants’ groups (residents and medical interns). (p = 0.55) and gender groups. (p = 0.09).

### Qualitative results

In this step, 17 participants contributed. The profile characteristic of the participants has shown in Table 2.

**Table 2:** Profile of participants in the qualitative step

Profile	Gender	Age	Academic level
1. Medical intern	Female	28	MD* student
2. Medical intern	Male	30	MD student
3. Medical intern	Female	28	MD student
4. Medical intern	Male	29	MD student
5. Medical intern	Male	30	MD student
6. Medical intern	Male	35	MD student
7. Medical intern	Female	27	MD student
8. Medical intern	Male	29	MD student
9. Medical intern	Female	24	MD student
10. Resident	Female	30	MD graduate
11. Resident	Male	36	MD graduate
12. Resident	Female	28	MD graduate
13. Resident	Female	27	MD graduate
14. Resident	Female	29	MD graduate
15. Resident	Male	32	MD graduate
16. Resident	Male	35	MD graduate
17. Resident	Female	34	MD graduate
*MD: Medical Doctor			

The qualitative results were explored in a theme entitled “internalized unprofessional culture.” This theme includes three categories “encouraging contextual risk factors towards unprofessionalism,” “suppressing unprofessionalism reporting,” and “disbelieving professionalism as a key responsibility.” (Table 3). The current results showed that the cultural components of the investigated context mainly promote unprofessionalism. The culture of a system formed a set of beliefs, values, attitudes, and experiences of the system members. The theme addressed three components of context, attitude, and beliefs of members and experiences of implicit encouraging factors that form the culture of unprofessionalism. The workers and learners in the educational system learned and conducted unprofessional behaviors in the healthcare service and taught others through role modeling. Gradually, these components were internalized into the system and formed by unprofessionalism culture in the system. The categories were explained below:

**Table 3:** The experiences of participants related to the factors affecting on their engagement in unprofessional behavior

Sub-category	Category	Theme
Negative role models	**Encouraging contextual risk factors towards unprofessionalism**	**Internalized unprofessional culture**
Encouragement of unprofessional behaviors		
Weakness of education		
Hierarchical relationship	**Suppressing of unprofessionalism reporting**	
Reception of negative feedback		
Deficiency of awareness of role and responsibility	**Disbelieving professionalism as a key responsibility**	
Non-commitment to professionalism principles		

### A) encouraging contextual risk factors toward unprofessionalism

This category discussed the contextual factors related to negative role models, encouragement of unprofessional behaviors in the system, and weakness of education. The participants stated that the frequent encounter of unprofessional behaviors of healthcare team members, including educators and workers, persuaded them to not adhere to professional principles. The self-profit, flying the patient to the physician’s private office, irresponsibility, and unpunctuality was explored as professionalism challenges. A resident expressed:

*The physicians prescribed medications to the patients in veins to charge a fee. (A 30-year-old female).*

*When overcrowding the clinics, the patients were flown as the outpatient in the physician*’*s office when they should be treated here. (A 36-year-old male resident).*

The weakness of the educational system and negative role models in informal education was explored in the category. A medical intern stated:

*Some team members were warning us about conducting unprofessional principles while we learned from them. (A 28- year-old female).*

*Our supervisors had conducted unprofessional behaviors such as requesting unnecessary para-clinic tests for the patient, not introducing themselves to the patient, and not wearing a medical gown at work. When we conducted these activities, members gave negative feedback to us while we learned from them how to act. (A 28-year-old female, Medical intern).*

A medical intern stated in the case of weakness of educational system:

*There was no specified definition and code of conduct for professional behavior. There hold no specific course for training professional behaviors. In addition, the unprofessional behaviors of physicians became common and usual for us. (A 30-year-old male).*

### B) suppressing unprofessionalism reporting

The participants believed various factors such as hierarchical relationships and the fear of upstream authorities resulted in the development of an unprofessional culture in the system. A medical intern stated:

*My friend and I prevent reporting practitioners’ errors since it will cause us trouble. (A 29-year-old male).*

A medical intern said:

*I observed many unprofessional behaviors, but less attention was paid to them. When I protested against the unprofessional behaviors, the practitioner quickly guarded against this protest. (A 30-year-old male).*

In the case of reception of negative feedback for the report of unprofessional behaviors, a medical intern stated:

*I made an error. Instead, I was given personal feedback, and I was reprimanded in front of others. I would never report my errors, anymore. (A 35-year-old male).*

### C) disbelieving professionalism as a key responsibility

Personal factors such as deficiency of awareness of role and responsibility, non-commitment to professionalism principles, and breaches of professionalism were explored in this category.

In the case of lack of awareness of role and responsibility, a resident stated:

*The lack of a job description for every team member causes I do not know what to do. (A 28-year-old female).*

In the case of a lack of perception of adherence to professional behaviors, a medical intern stated:

*I think introducing a role of a healthcare provider to the patient is unnecessary since the patients did not perceive the difference between a resident and an intern. (A 27-year-old female).*

*When the patients had no medical knowledge, there was no need to describe the medical measures to them. (A medical intern, 29-year-old male).*

*I did not introduce myself to the patient due to fatigue or excessive work. (A 27-year-old female, A resident).*

In the case of professionalism breaches, a resident stated:

*The confidentiality of the patients*’ *information was not observed thoroughly. The patients*’ *information was readily available to others. (A 29-year-old female).*

In the case of non-commitment to professional dressing, a medical intern stated:

*My coworkers did not use appropriate equipment such as masks and shields because the protection equipment was cumbersome. They could not stand using such tools for long shifts. (A 24-year-old female).*

## Discussion

The results showed the proportion of participants who engaged in unprofessional behavior more than those who did not participate. Moreover, the results confirmed that although the participants stated that these behaviors were unprofessional, those who participated in the following unprofessional behaviors were more than those who did not. The highest ratio of engagement in unprofessional behavior of the participants was reported in ‘failure to introduce yourself and nurses and physician assistants to the patient and his family’. The qualitative results explored the reasons for the participants’ engagements in unprofessional behaviors in the theme entitled “internalized unprofessional culture,” including three categories of **“**encouraging contextual risk factors towards unprofessionalism,” “suppressing of unprofessionalism reporting,” and “disbelieving professionalism as a key responsibility.”

The quantitative results showed that more participants were involved in unprofessional behaviors, which were not significantly different among groups of residents and medical interns. The results were explained by internalized unprofessional culture in the qualitative phase. The contextual risk factors, such as a weakness in teaching professionalism, negative role modeling, encouragement towards non-adherence of professionalism, and implicit education of unprofessional behaviors influenced the engagement of unprofessional behaviors among the residents and medical interns. In addition, the deficiency of participants in competencies of professionalism and non-commitment to these principles were explored as reasons for engaging the participants in unprofessional behaviors. According to the qualitative results, the weakness of the educational system in the teaching of professional principles in the explicit (formal and informal curriculum) and hidden curriculum, and failure to accept professionalism as a requirement resulted in the participants could not achieve the key ability to make decisions and conduction of professional behavior. In addition, the participants worked in a system that promoted unprofessionalism and observed the unprofessional behaviors of others, so most people experienced engagement in unprofessional behaviors. The quantitative results confirmed more participants engaged in unprofessional behaviors. Similarly, Reddy and colleagues showed that unprofessional behavior emerged from “professionalism breaches (such as poor role model behavior, lack of patient care focus, and disregard for student needs) and overt hostility towards professionalism education” [31]. In line with our results, Mak-van der Vossen classified factors involved in the occurrence of unprofessional behavior into four categories: contextual factors (unclear standards, learning environment that did not encourage professionalism, inadequate supervision, poor role modeling, and culture that rewards unprofessional behavior), personal factors (competency deficits, learning disabilities), external factors (psychosocial stressor, financial challenges) and interpersonal factors (different cultural expectations, hierarchy, poor understanding of roles and responsibilities) [32]. Likewise, the explored reasons for participants’ engagement in unprofessional behaviors were classified into contextual, interpersonal, and personal levels in our study. The findings of various studies indicated that the complex inter-relationship issues at multiple interpersonal, individual, and organizational levels formed the phenomenon of unprofessional behavior [33–38]. Studies mentioned weaknesses of the evaluation system, and allocation of insufficient time to educate and evaluate professionalism influenced increasing unprofessional behaviors [39–41].

The quantitative results showed despite the participants acknowledging that most behaviors were unprofessional, those who engaged in these behaviors were more than those who did not participate. The results mean the participants understood that the behaviors were unprofessional but conducted them. The explored results in the qualitative step justified the occurrence of unprofessional behavior among the participants. The findings indicated that unprofessional behaviors were internalized in the culture of the system. The category of encouraging contextual risk factors addressed the factors such as negative role models and the ignorance of unprofessional behaviors in the workplace that influenced the results. Frequent observation and encounters with unprofessional behaviors by different members of the healthcare system resulted in such behaviors being normalized for the participants. In addition, the category of “suppressing unprofessionalism reporting,” addressed the hidden factors that encouraged the participants to neglect the unprofessional behaviors, and normalized their engagement in them. The participants believed they learned to ignore the unprofessionalism in the system and take no action for reporting them. These risk factors eliminated the participants’ sensitivity to unprofessional behaviors and their commitment to adhere to professional principles. These may result in the participants recognizing the unprofessional behaviors but engaging with them. Thus, encouraging unprofessional behavior and suppressing unprofessional reporting assisted in the formation of unprofessional culture in the system. In line with our results, Pavithra and colleagues indicated that the weakness of the organization to address unprofessional behaviors significantly impacts the internalization of unprofessional behaviors as professional norms. These issues led to ineffective remediation of these behaviors [42]. They revealed the multifaceted interaction issues between organizational and individual factors that influenced the occurrence of unprofessional behaviors among healthcare workers. Studies elucidate that contextual, organizational, and socio-cultural factors (such as negative, internalized sub-cultures, and prevalent incivility) substantially influenced the experience of unprofessional behaviors of workers [42–47].

The results showed four behaviors that the participants acknowledged being unprofessional, and they engaged significantly, were classed into two domains: failure to comply with regulations in the organization and working process (i.e., eating or drinking in the clinic hallway) and negligent of the team and system duties. The highest ratio of participants’ engagement in unprofessional behavior was reported in ‘failure to introduce yourself and nurses and physician assistants to the patient and his family.’ The qualitative results indicated these issues may result from the problem of the educational system, cultural factors, and personal issues. The categories of disbelieving professionalism as a key responsibility and encouraging contextual risk factors were explained in the achieved findings. The personal issues related to the recognition and commitment to the professionalism principles, deficiency of recognition of professional roles, and team responsibilities, and failure to conduct them were explored in the category of ‘disbelieving professionalism as a key responsibility’. In addition, lack of compliance with regulations and obligations of duties is caused by cultural factors. Moreover, the cultural factors of physician-centeredness may affect non-compliance with the team’s regulations mentioned in the studied context. This issue was discussed in different studies in our context [48, 49]. Medical interns and residents and considered themselves superordinate in the healthcare system. They learned not to accept membership in a team and a system and not obey the rules in the physician-centered climate. This issue is an essential obstacle to forming an identity as a professional and interprofessional collaborator. In line with our results, Keshmiri’s results showed “uni-professional centrism” as a barrier to forming an interprofessional identity of healthcare team members and neglecting team responsibilities [50]. Similar to the explored factors in our study, competency deficits, poor understanding of roles and responsibilities, poor role modeling, ‌rewarding unprofessional behaviors, and non-commitment to professional principles were explained as risk factors of unprofessional behavior development [51, 52]. The complex relationship between personal and systemic factors in the occurrence of unprofessional behaviors was mentioned in different studies [42, 44–47).

More than the males, the female participants identified the behaviors as unprofessional. In the qualitative phase, the participants acknowledged that females were more sensitive to unprofessional behaviors due to greater caution and empathy. Similar to our results, Elger showed that female physicians were better able to correctly identify unprofessional behaviors, including those related to patient information confidentiality [53]. The findings of Nath’s study showed that females were more inclined to label behaviors as unprofessional in West Virginia [54]. Likewise, there was a significant difference between males and females in terms of sensitivity to unprofessional behaviors in different dimensions, such as altruism and respect [55, 56].

### Limitation

The generalizability of the results is constrained due to the limited sample size. Self-reported instrument in the quantitative step was a limitation of the present study. In addition, the qualitative findings may not apply to other populations with different cultural backgrounds.

## Conclusion

The results showed that in most behaviors, although the participants acknowledged that the particular behaviors were unprofessional, the participants who engaged in these behaviors were more than those who did not participate. This resulted from the internalized unprofessional culture in the workplace. The findings indicated the personal factor of disbelieving professionalism as a key responsibility and systemic factors including encouraging contextual risk factors and suppressing unprofessionalism reporting encouraged the participants to neglect the professional behaviors and normalized the engagement in unprofessionalism.

Recognition of the nature and extent of students’ unprofessional behaviors facilitates educational discussion of these behaviors among teachers and students. The results might assist in establishing an assessment system and feedback mechanism to solve the problem of “failure to fail” problem. In addition, these results provide medical educators insights into the development of professional courses that equip learners with adherence to professionalism and coping skills to deal with unprofessionalism in the healthcare system.

## Data Availability

The datasets generated during and analyzed during the current study are not publicly available due to the confidentiality of data but are available from the corresponding author at a reasonable request.
